# Malarial Antibodies and Endemicity: Does It Affect SARS-CoV-2 Severity and Outcomes?

**DOI:** 10.7759/cureus.46871

**Published:** 2023-10-11

**Authors:** Prayas Sethi, Tamoghna Ghosh, Souradeep Chowdhury, Raunak Bir, Nishant Verma, Shivam Pandey, Arulselvi Subramanian, Ved Meena, Neeraj Nischal, Sulagna Bhattacharjee, Ajisha Aravindan, Rahul K Anand, Devalina Goswami, Richa Aggarwal, Naveet Wig

**Affiliations:** 1 Medicine, All India Institute of Medical Sciences, New Delhi, IND; 2 Infectious Diseases, All India Institute of Medical Sciences, New Delhi, IND; 3 Microbiology, Employees’ State Insurance Corporation (ESIC) Medical College and Hospital, Faridabad, IND; 4 Microbiology, All India Institute of Medical Sciences, New Delhi, IND; 5 Biostatistics, All India Institute of Medical Sciences, New Delhi, IND; 6 Laboratory Medicine, All India Institute of Medical Sciences, New Delhi, IND; 7 Anesthesiology, Pain Medicine, and Critical Care, All India Institute of Medical Sciences, New Delhi, IND

**Keywords:** severity, mortality, malaria, covid-19, antimalarial antibody

## Abstract

Background

India has a disproportionately lower rate of coronavirus disease 2019 (COVID-19) severe disease and lower death rates with respect to other parts of the world. It has been proposed that malaria-endemic countries such as India are relatively protected against severe COVID-19 disease and deaths.

Methods

This was a cross-sectional, analytical, observational study conducted from August 2020 to July 2021 at a tertiary care COVID-19-designated center in New Delhi, India. It aimed to study the association between antimalarial antibody levels and COVID-19 disease severity and outcomes.

Results

One hundred forty-six patients were included in the final analysis. The mean (standard deviation {SD}) age of the study population was 44.6 (17.2) years, and there were 85 (58.2%) males. Sixty-five patients had mild disease, 14 patients had moderate disease, and 67 patients had severe disease at the time of enrolment in the study. Forty-six patients expired during the hospital stay. For the antimalarial antibody, there was a statistically significant difference between mild and moderate (p=0.018), mild and severe (p=0.016), and mild and combined moderate and severe diseases (p=0.013). However, there was no difference between the patients who survived and those who did not.

Conclusion

Antimalarial antibody levels may not be associated with the outcomes of COVID-19 during hospital stay. However, this study has provided some insights into the relationship between the severity and outcomes of COVID-19 and the levels of antimalarial antibodies.

## Introduction

Severe acute respiratory syndrome coronavirus 2 (SARS-CoV-2), which causes coronavirus disease 2019 (COVID-19), has rapidly evolved into a pandemic, affecting around 673 million people globally and causing around 6.7 million deaths. In India, it has affected more than 44.9 million people and led to more than half a million deaths, as of September 2023 [1]. Although it was initially characterized as a respiratory system infection, it is now considered a systemic disease with the involvement of multiple organs [2-4]. It has led to the derangement of hematological, immunological, inflammatory, and biochemical parameters both in the young and in the old [5,6].

India has a disproportionately lower rate of COVID-19 severe disease and lower death rates with respect to other parts of the world [6]. The situation mirrors that of Africa, as has been found in a recent meta-analysis [7]. A number of factors have been proposed to be contributing to this variation, including administrative and governance factors, climate, immunity, and genetic factors. However, India has a disproportionately higher burden of other infectious diseases such as malaria, tuberculosis, and HIV/AIDS [8]. India alone contributes 77% of the total malaria cases in Southeast Asia [9]. It has been proposed that malaria-endemic countries such as India are relatively protected against severe COVID-19 disease and deaths, as during the early parts of the COVID-19 pandemic, a negative correlation was found between malarial endemicity and COVID-19 mortality [10]. An ecological study has also suggested that malaria prevalence is negatively correlated with COVID-19 mortality [11]. An Indian study put forth a similar hypothesis where the annual parasite index from 30 districts in Odisha state of India from 2010 to 2019 was found to be inversely related to COVID-19 cases until July 2020 [10]. Another study from India has also demonstrated significantly faster clearance of COVID-19 infection in patients coinfected with malaria [12].

The induction of interferons and neutralizing antibodies due to chronic infection by *Plasmodium falciparum*, polymorphisms in the angiotensin-converting enzyme 2 (ACE2), cluster of differentiation 147 (CD147)-mediated entry of SARS-CoV-2 and *P. falciparum* into host cells, and the recognition of malarial glycosylphosphatidylinositol (GPI) antibodies by COVID-19 membrane glycoproteins (GPs), spike GPs and GPs that have acetyl esterase and hemagglutination properties are some of the pathogenic mechanisms implicated in the lower incidence or severity of COVID-19 in patients with malarial antibodies [13-20]. Several quinoline analogues, including chloroquine, hydroxychloroquine, amodiaquine, ferroquine, and mefloquine, exhibit broad anti-coronavirus activity in vitro [21]. Our study proposed to study the association between the severity and outcome of COVID-19 infection and the antimalarial antibody levels.

## Materials and methods

Study population

This was a cross-sectional, analytical study conducted from August 2020 to July 2021 at a single tertiary care COVID-19-designated center in New Delhi, India. It aimed to study the association between antimalarial antibody levels and COVID-19 disease severity and outcomes. Adult (age of >18 years) subjects with a positive reverse transcription-polymerase chain reaction (RT-PCR) for the severe acute respiratory syndrome coronavirus 2 (SARS-CoV-2) from the secretions of the upper or lower respiratory tract were eligible for inclusion in the study. Tests for SARS-CoV-2 were performed as per the Indian Council of Medical Research (ICMR) guidelines. Disease severity was classified as per the Indian Ministry of Health and Family Welfare (MoHFW) COVID-19 management guidelines: patients were deemed to have severe disease if they had shock or their oxygen saturation (SpO_2_) was less than 90% or their respiratory rate was greater than 30 per minute (necessitating invasive or noninvasive mechanical ventilation); moderate disease if they had dyspnea, SpO_2_ between 90% and 94%, or respiratory rate between 24 and 30 breaths per minute; and mild disease if there was no evidence of breathlessness or hypoxia (normal oxygen saturation). Informed consent was obtained from the patients before enrolling them in the study. We excluded patients who refused to give consent or were below 18 years of age. The study was approved by the Institutional Ethics Committee of the All India Institute of Medical Sciences.

Sample size

A formal sample size calculation was not done, as it was a novel study, and there was no robust data on the prevalence of malarial antibodies available among the general population in India. Instead, a convenient sample of 150 was taken (based on available resources and funding), aiming to achieve nearly equal patients in mild and moderate to severe disease categories. Consecutive COVID-19-positive patients presenting to the hospital were recruited.

Data collection

Data was recorded on a predesigned proforma that included patients’ demographic details, comorbidities, presenting symptoms, duration of illness, severity of the illness, and laboratory parameters (complete blood count, liver function test, and D-dimer). A blood sample was taken for the estimation of antimalarial antibody levels. Patients’ sera were tested for the presence of IgG antimalarial antibodies with the quantitative estimation of the levels using a commercially available enzyme-linked immunosorbent assay (ELISA) kit. Wherever clinically suspected, a concomitant active malaria was evaluated with peripheral blood smear (PBS) for malarial parasite (thick and thin smears), quantitative buffy coat assay, and malaria antigen test pan lactate dehydrogenase (LDH)/*Plasmodium falciparum* histidine-rich protein 2 (Pf HRP2).

Statistical analysis

Quantitative variables were checked for normality by the Shapiro-Wilk test. Variables following normal distribution were expressed as mean (standard deviation {SD}), and variables that followed skewed distribution were expressed as median (interquartile range {IQR}). Means were compared using Student’s t-test and medians using the Mann-Whitney U test. Categorical variables were expressed as frequency (percentage). The chi-square test was used for categorical data. Statistical significance was determined at a p-value of <0.05. Data was analyzed using Stata Release 14 (2015) (StataCorp LLC, College Station, Texas).

## Results

Demographics and patient characteristics

A total of 154 patients were screened for possible inclusion in the study. Among them, seven patients refused to give consent, and 147 patients were included in the final analysis. The mean (SD) age of the study population was 44.2 (17.5) years, and there were 86 (58.5%) males. Sixty-six patients had mild disease, 14 patients had moderate disease, and 67 patients had severe disease at the time of enrolment in the study. The outcome data of 142 patients was available as five patients either got transferred to a step-down hospital/nursing home or were discharged on oxygen as per patients’ request. Forty-six patients expired during the hospital stay, and 96 patients were discharged. Of the 46 patients who expired, all of them had severe disease, and 26 (56.5%) of them were males. No significant difference was observed in the outcome of patients based on sex. However, there was a statistically significant difference in hemoglobin level, total and differential leukocyte counts, platelet count, aspartate transaminase (AST), alanine transaminase (ALT), prothrombin time, and D-dimer levels between the survivors and non-survivors as depicted in Table 1.

**Table 1:** Comparing parameters between expired and improved patients. The values are represented as mean±SD or median (IQR). *Statistically significant SD, standard deviation; IQR, interquartile range; AST, aspartate transaminase; ALT, alanine transaminase; PT, prothrombin time

	Expired Group (n=46)	Improved Group (n=96)	P-Value
Age (years)	52.2±18.0	40.1±15.9	<0.001*
Hemoglobin (g/dL)	9.3±2.3	12.3±2.9	<0.001*
Total Leukocyte Count (>1000/mm^3^)	17.4±9.7	8.3±4.3	<0.001*
Neutrophils (%)	89.3±7.1	63.7±15.6	<0.001*
Lymphocytes (%)	6.8±4.7	25.2±12.3	<0.001*
Neutrophil-Lymphocyte Ratio	20.8±15.6	5.1±7.3	<0.001*
Platelet Count (>1000/mm^3^)	146.2±117.2	234.2±106.9	<0.001*
AST (IU/L)	53 (33-94)	29 (20-42)	<0.001*
ALT (IU/L)	34 (18-65)	25 (18-53)	0.522
PT (seconds)	21.6±11.1	14.8±4.4	<0.001*
D-Dimer (ng/dL)	3.4 (2.4-5.6)	1.0 (0.4-2.9)	0.003*

Malarial antibody levels

**Figure 1:**
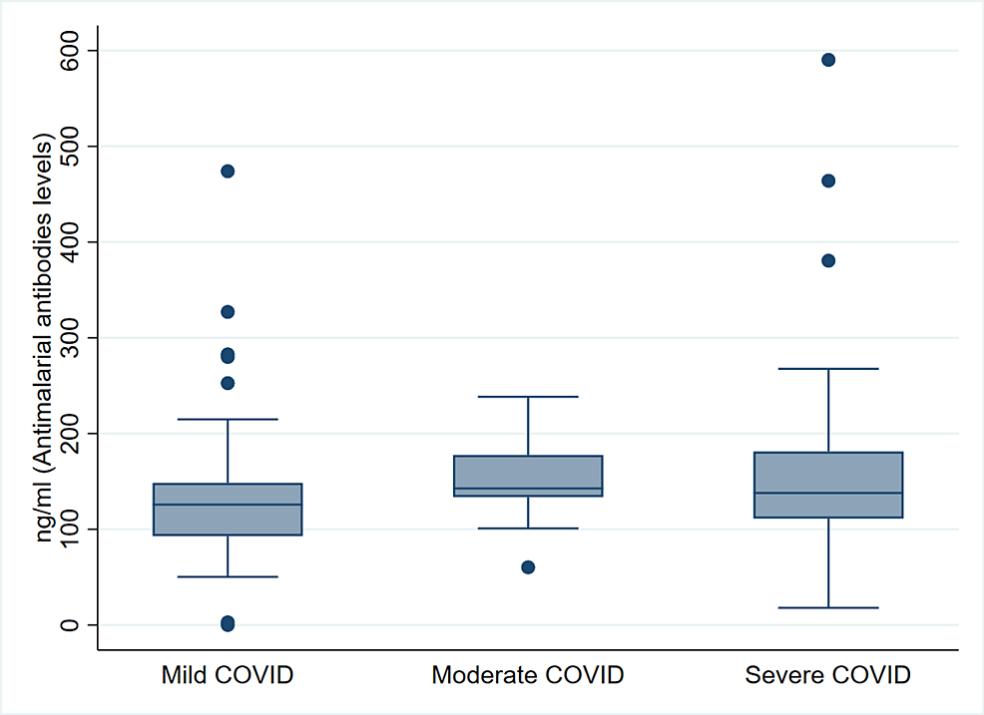
Antimalarial antibody levels (median {IQR}) among the mild, moderate, and severe disease patients of COVID-19. IQR, interquartile range; COVID-19, coronavirus disease 2019

The median (IQR) value of antimalarial antibody levels in the entire cohort of patients was 133.4 (100.9-167.0) ng/mL and for mild, moderate, and severe diseases, it was 125.8 (92.9-148.4), 142.7 (133.7-177.6), and 137.9 (111.2-181.2) ng/mL, respectively, as illustrated in Figure 1.

**Figure 2:**
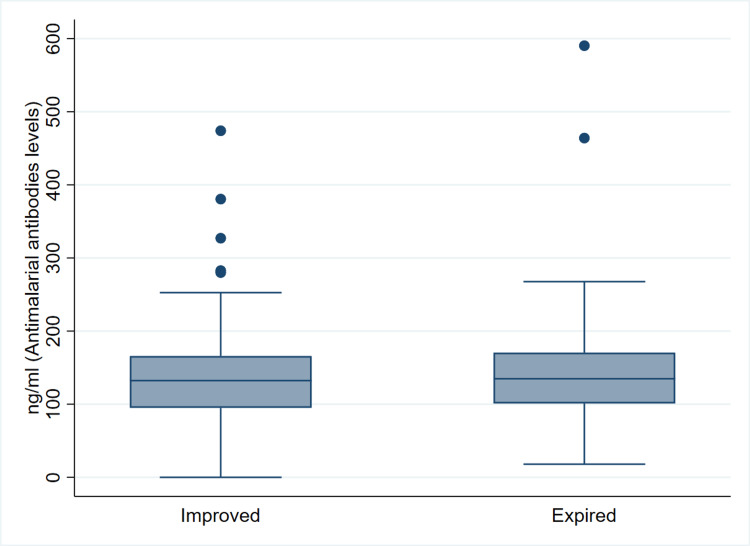
Antimalarial antibody levels (median {IQR}) among the patients of COVID-19 according to outcomes. IQR, interquartile range; COVID-19, coronavirus disease 2019

There was a statistically significant difference between mild and moderate (p=0.038) and mild and combined moderate and severe diseases (125.8 {92.9-148.4} versus 140.3 {113.4-179.1}) (p=0.021) but not between mild and severe diseases (p=0.053), though there was a trend toward significance. The median (IQR) values of antimalarial antibody levels in the improved and expired patients were 132.3 (94.9-165.9) and 134.8 (100.9-170.5) ng/mL, respectively, but there was no statistically significant difference (p=0.619), as shown in Figure 2.

**Figure 3:**
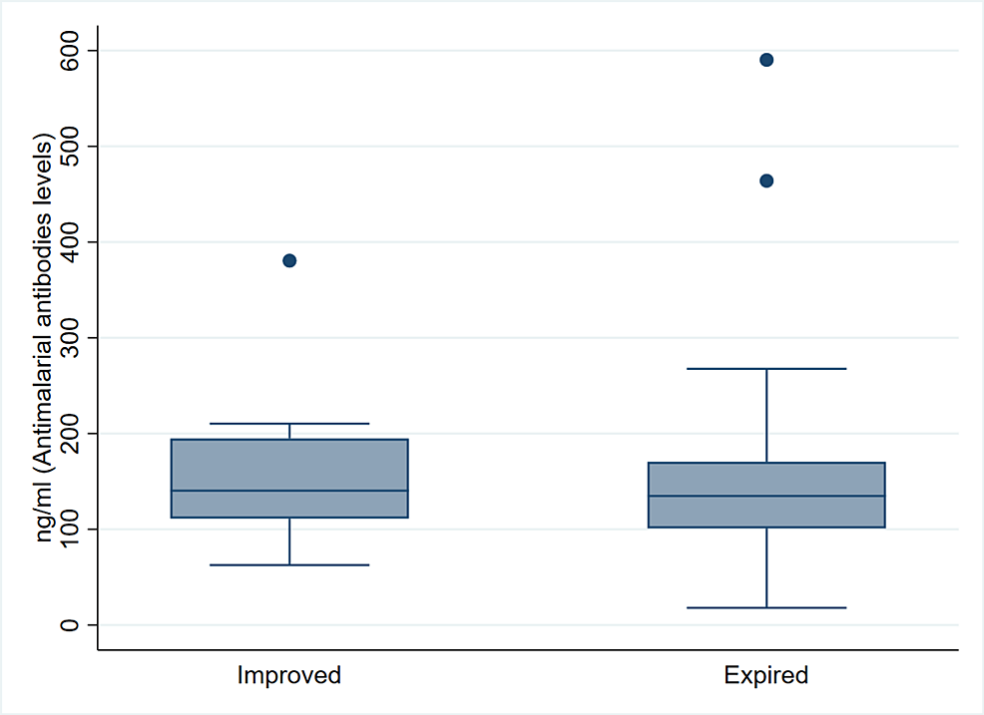
Antimalarial antibody levels (median {IQR}) among the patients of COVID-19 according to outcomes, in the severe disease category. IQR, interquartile range; COVID-19, coronavirus disease 2019

Since all the deaths were in the severe disease category, we compared the antimalarial antibody levels between the survivors and non-survivors with severe disease. The median (IQR) values in the improved and expired patients were 140.3 (111.2-194.8) ng/mL and 134.8 (100.9-170.5) ng/mL, respectively, and the difference was statistically not significant (p=0.403) as shown in Figure 3.

The median (IQR) values of antimalarial antibody levels in the improved patients among severe disease were 140.3 (111.2-194.8) ng/mL, and in mild patients where all improved, it was 125.8 (92.9-148.4) ng/mL. This difference was statistically significant (p=0.011).

The median (IQR) values of antimalarial antibody levels in male and female patients were 132.7 (111.2-164.8) and 137.9 (94.5-173.9) ng/mL, respectively. However, there was no statistically significant difference between males and females (p=0.812).

Logistic regression

On logistic regression analysis, antimalarial antibody levels were not significantly associated with mortality on univariate or multivariate analysis and on adjusting for complete blood count (hemoglobin concentration, total leukocyte count, and platelet count), liver function test (AST and ALT), and D-dimer.

## Discussion

Our study has shown that malarial antibodies may not have an impact on the severity and outcomes of COVID-19. However, some statistical significance has been observed showing higher malarial antibody levels in patients with severe disease. Likewise, significantly higher antibody levels have been found in patients who improved among the severe disease group versus the mild group.

It has been hypothesized that persons living in malaria-endemic areas acquire repeated subclinical infections and might possess immunity, which can be beneficial in mounting successful immune responses against other pathogens such as SARS-CoV-2 [22]. Though malaria influences adaptive immunity, its impact is felt more with the activation of innate immunity resulting in both trained immunity and immune tolerance, both of which are beneficial in protecting against severe malaria and may offer cross-protection against other diseases such as COVID-19 [23,24].

A study from a malaria-endemic country, Uganda, found that those with low previous *P. falciparum* exposure still had a higher proportion of cases of severe or critical COVID-19 than those with high *P. falciparum* exposure [25].

Our study has shown higher levels of antimalarial antibodies in patients with severe and moderate disease as compared to mild or asymptomatic COVID-19 disease. This is contrary to the proposed hypothesis of higher malarial antibody levels leading to protection against severe disease [22]. However, the higher antibody levels among patients with severe disease in our study may be possible because of severe disease generating higher immune response and its sero-reactivity against malaria. A relationship between increased SARS-CoV-2 sero-reactivity and antimalarial humoral immunity has been demonstrated [26]. However, cross-reactive anti-malarial antibodies did not demonstrate significant neutralizing activity via invasion at any dilution with the SARS-CoV-2 virus [27]. Another hypothesis possible with the results and trends of statistical significance is that the disease severity may be higher in those with higher levels of antimalarial antibodies. However, the level of protection against mortality also may be higher in patients with higher anti-malarial antibodies. Clinically, it means that malaria-endemic regions may have a considerable burden of severe disease but with less unfavorable outcomes.

On the analysis of the outcome in terms of death versus discharge from the hospital, patients who expired had higher antimalarial antibody levels as compared to patients who improved and were discharged, but the difference was not statistically significant. This may be because mortalities happen with severe disease only, hence the similar results. To further clear this, we also analyzed the antimalarial antibody levels among severe disease patients who expired and improved. There was no statistical difference between the two. However, there was a trend toward significance in the comparison of patients who improved overall to the patients who improved among the severe disease patients and in the comparison of patients who expired to those who improved among the severe disease patients. This emphasizes the fact that malarial antibodies may not be protective against severe disease and poor outcome, but among patients with severe disease, antimalarial antibodies may have a better outcome. In an epidemiological study by Anyanwu, malaria prevalence, life expectancy, and mortality rate were significantly associated with COVID-19-infected patients on multivariate regression analysis [11].

In our study, antimalarial antibody levels were not significantly associated with mortality on univariate or multivariate analysis after adjusting with other variables. The innate immune response against malaria includes natural killer cells, monocytes, macrophages, and pro- and anti-inflammatory cytokines [28,29]. Thus, a person with malarial immunity acquired due to previously repeated sub-infections may mount an initial immune response after exposure to SARS-CoV-2, but this is a nonspecific immune response and may not be effective. Thus, antimalarial antibodies may not be protective against COVID-19 mortality.

However, there were a few limitations of this study; the sample size was small, particularly for the patients who expired, to definitively conclude the findings. The follow-up of the patients was until discharge or death from the hospital; delayed mortality was not considered. The kits that were used are not routinely used for clinical purposes. They were imported from the USA. The accuracy of the kits could not be ascertained. Some other potential bias was regarding the recruitment of patients. They were enrolled in the study not on the day of presentation but on any random day of hospital stay.

## Conclusions

Antimalarial antibody levels may not be associated with the outcomes of COVID-19 during hospital stay. However, median antimalarial antibody levels have been significantly higher in patients with severe disease on univariate analysis and among patients with severe disease; patients who improved had higher mean and median levels of malarial antibody levels as compared to those who expired. Also, patients who improved among the severe disease group had higher mean antimalarial antibody levels compared to those who had mild to moderate disease. So, whether or not SARS-CoV-2 generates an antimalarial antibody immune response, which is proportional to disease severity, or patients with heightened antimalarial antibody levels had a higher propensity for severe disease may be an area to be explored. Also, protection against mortality among patients with severe disease may be explored as a possible cause of malarial endemicity as a protective factor against SARS-CoV-2 mortality. Further studies with larger sample sizes and diverse populations may be needed for these issues.

The study provides valuable insights into the relationship between antimalarial antibodies and COVID-19 outcomes, which could be significant in regions such as India with a high burden of both malaria and COVID-19. The study uses a relatively large sample size for a single-center study and covers a reasonable time frame. The data collection and statistical analysis appear to be thorough and well-documented. However, the findings should be interpreted cautiously due to the limitations mentioned above, and further research with larger and more diverse populations is warranted to confirm and better understand these associations.
